# Estimated number of seriously injured road users admitted to hospital in France between 2010 and 2017, based on medico-administrative data

**DOI:** 10.1186/s12889-021-10437-0

**Published:** 2021-03-08

**Authors:** Anthony Zullo, Maxime Large, Emmanuelle Amoros, Jean-Louis Martin

**Affiliations:** Univ Gustave Eiffel, Univ Lyon, Université Lyon 1, IFSTTAR, UMRESTTE UMR_T 9405, 25 avenue François Mitterrand, Bron, 69675 France

**Keywords:** Road accident, Hospital discharge registry, Serious injury, External cause of morbidity/mortality, Under-recording, ICD classification, AIS classification

## Abstract

**Background:**

In France, like in most developed countries, the number of road accident fatalities is estimated from police data. These estimates are considered to be good-quality, unlike estimates of road injuries admitted to hospital, and especially serious injuries.

**Methods:**

The present study aimed to supply such data from French hospital medical information data-bases (PMSI). In the PMSI data-bases, road accident victims are identified by external causes of morbidity and mortality, which should be systematically recorded in case of injury, but are often missing. We therefore modeled presence/absence of external cause from the relevant subset of the medicine-surgery-obstetrics PMSI data-base using a logistic regression, and then weighting the results by inverse estimated probability. As ICD-10 coding does not include injury severity, we used the AAAM10 conversion instrument developed by the American Association for Automotive Medicine, originators of the Abbreviated Injury Scale, so as to conform to the European Commission’s definition of serious injury.

**Results:**

The number of road-accident related hospital admissions is estimated to be about 100000 per year; serious injuries increased from about 18000 in 2010 to almost 20000 in 2017, with almost 17000 in 2012 and 2013, with a mean of one fatality per 5 serious injury admissions.

**Conclusions:**

These serious injury estimates are close to those obtained by our team from other data and with a different estimation method. The present method has the advantage of using ICD codes for injured people admitted to hospital. This classification and data source (hospital discharge registry) are also used by most european countries reporting serious injury estimates to the Commission. It allows cost estimation of hospital care, and could be applied to other types of accidental injury.

**Supplementary Information:**

The online version contains supplementary material available at (10.1186/s12889-021-10437-0).

## Background

In France, like most developed countries, statistics for road-accident casualties and fatalities come from police data. These are exhaustive for deaths, but several studies clearly showed that serious injury is underestimated and subject to several biases [1, 2]. For example, cyclists and motorcyclists are under-counted when no third party is involved in the accident. Such recording bias can seriously affect road safety issues.

Police data also fail to report precise health status. In the French data, this is reduced to 3 categories: death (within 30 days), injured admitted to hospital, injured not admitted to hospital.

To avoid differential underestimation and correctly assess severity, information is needed from the medical structures treating the victims. Most developed countries assess serious injury only on the basis of hospital discharge registries. Diseases and injuries are described with the International Statistical Classification of Diseases and Related Health Problems (ICD), revision 9 or 10. For injury, a supplementary code indicates the cause (external cause of morbidity and mortality), allowing road-accident victims to be distinguished. However, ICD coding does not include severity level per lesion, unlike the Abbreviated Injury Scale (AIS) [3], developed by the American Association for Automotive Medicine (AAAM), which details injury assessment very precisely, associating each lesion to a level of immediate severity from 1 (minor) to 6 (maximal).

The European Commission uses the AIS in its definition of “serious road injury” as comprising at least one level ≥3 injury (MAIS3+), and now requires member states to report their number.

To implement this definition with ICD coding, a correspondence matrix between ICD and AIS is provided by the AAAM [4, 5], estimating the severity level of each ICD code as AIS level ≥3 or <3.

In France, another very specific method has been used to produce estimates of serious injury [6, 7]. For purposes of international comparison, however, as in the SafetyCube project [8, 9], it would be very helpful to use a method closer to that used elsewhere, based on national discharge hospital data. This could also reinforce the method currently used, which involves more precise information on crash circumstances than is found in hospital records.

The main aim of the present study was to estimate the number of serious road-accident casualties admitted to hospital, based on the French national PMSI medico-administrative data-base. The secondary objective was to estimate the number of injuries of whatever severity sustained under the same circumstances, based on the same data. These estimates were then compared to those obtained with the specific French methodology referred to above.

In order to achieve these two objectives, we need to address two problems. The first one is the lack of external causes of morbidity/mortality recording in hospital discharge registries. While the report of injuries is systematic because mandatory, it is not the same for the external causes of morbidity/mortality. Being not relevant from an economical point of view, these external causes are recorded in only 28% of cases on average. This low percentage is a data quality issue. The second problem to address is the necessity to have a severity scale associated with each trauma in order to be able to identify serious injuries. The ICD coding lacking of such severity scale, we need to map it into another injury coding system like the AIS. However, the equivalence between ICD codes and MAIS level ≥3 can only be approximate; several ICD codes can lead to the same AIS code, while a single ICD code can potentially have several corresponding AIS codes.

## Methods

### Data

The study was based on data from the PMSI data-base, a medical information system describing and costing hospital activity [10]. It provides a precise description of the medical activity of each health center, and measures costs related to the various pathologies in terms of diagnosis-related groups.

The PMSI comprises 4 fields: medicine-surgery-obstetrics-odontology (PMSI-MCO), after-care and rehabilitation (PMSI-SSR), medicine-psychiatry (RIM-P), and home hospitalization (PMSI-HAD). For the present purpose of estimating serious road casualties admitted to hospital in France between 2010 and 2017, only the PMSI-MCO data were relevant.

To validate our method, we also used data from the Rhône road trauma Registry, an almost exhaustive continuous record of all victims of road accidents in the Rhône administrative *Département* of France (pop. 1.6m). The Registry is based on a network of all hospital services liable to be involved in the management of victims of road accidents in the area, including services in neighboring *Départements*. The hospital services range from emergency units, resuscitation unit, surgery, …to rehabilitation, and the forensic institute. These services provide notification forms (either paper or electronic) directly to the Rhône road trauma Registry. It includes people only treated at the emergency units, those admitted, and fatalities. Each individual lesion is coded into an AIS code, enabling the definition of “serious injury” to be applied directly.

#### Description of medico-administrative data

PMSI-MCO collates standardized discharge summaries per hospital stay, each of which summarizes one or more medical unit reports corresponding to stay in a given department, including admission and discharge/transfer. The discharge summaries are rendered anonymous by hash coding, and are then known as “anonymous discharge summaries”, each at least comprising a main diagnosis, underlying admission to the medical department, and possibly a related diagnosis (related to the main diagnosis) and one or more associated diagnoses. Diagnoses are currently coded according to ICD-10 [11]. For injuries, associated diagnoses beginning with V, W, X or Y provide information on the external cause of morbidity/mortality: i.e., the circumstances leading to admission. This includes whether the injury was intentional or not and, if accidental, whether it was a home and leisure injury, occupational injury or road injury. A Table presenting ICD-10 groups of external causes is shown as an additional material [see Additional file 1].

The present study concerned only admissions with at least one diagnosis of injury other than sequelae (ICD-10 codes S00 to T88 inclusive). This diagnosis family systematically involves an external cause. However, this cause is of no economic relevance, and is recorded in only 22-34% of cases. It is for this reason that we developed the estimation methodology presented in this article. Reporting rates of external causes of morbidity/mortality per year, in percentage stays for injury (excluding sequelae) are shown as an additional material [see Additional file 2].

The PMSI-MCO data used in the study were provided by the Hospital Information Technical Agency (ATIH). Table 1 lists the PMSI-MCO variables serving directly or indirectly in the study (source: ATIH).

**Table 1:** PMSI-MCO variables used in the study (source: ATIH)

PMSI variable	Description
anonyme	Anonymized ID
sexe	Gender
age	Age
modeEntree	Type of admission
modeSortie	Type of discharge
mois	Month of discharge
duree	Length of hospital stay
nbacte	Number of acts classifying the stay
nbrum	Number of medical unit reports
ghm2	Diagnosis-related group
ghs	Stay-related group
tarif	Price associated to stay-related group
diag	Diagnoses (main diagnosis, related diagnosis, associated diagnoses)
finess	Health establishment ID number
secteur	Health establishment sector (public or private)
categ_detail	Health establishment detailed category

#### Processing of medico-administrative data

This section describes the processing of the raw ATIH data. In the anonymous discharge summaries, the diagnosis maximizing the allocated yearly budget tends to be chosen by the center as the main diagnosis, other diagnoses being counted as “related” or “associated”. This is due to the current activity-based funding system, introduced in 2004 for public-sector hospitals and in 2005 for private-sector hospitals. Given the study objective, this differentiation is irrelevant. Table 2 shows ICD-10 injury groups (other than sequelae).

**Table 2:** ICD-10 injury groups

ICD-10 codes	Category
S00-S09	Injuries to the head
S10-S19	Injuries to the neck
S20-S29	Injuries to the thorax
S30-S39	Injuries to the abdomen, lower back, lumbar spine and pelvis
S40-S49	Injuries to the shoulder and upper arm
S50-S59	Injuries to the elbow and forearm
S60-S69	Injuries to the wrist and hand
S70-S79	Injuries to the hip and thigh
S80-S89	Injuries to the knee and lower leg
S90-S99	Injuries to the ankle and foot
T00-T07	Injuries involving multiple body regions
T08-T14	Injuries to unspecified part of trunk, limb or body region
T15-T19	Effects of foreign body entering through natural orifice
T20-T32	Burns and corrosions
T33-T35	Frostbite
T36-T50	Poisoning by drugs, medicaments and biological substances
T51-T65	Toxic effects of substances chiefly nonmedicinal as to source
T66-T78	Other and unspecified effects of external causes
T79	Certain early complications of trauma
T80-T88	Complications of surgical and medical care, not elsewhere classified

Our aim was to estimate as precisely as possible the probability of an external cause being recorded, independently of the type of cause. As injury necessarily implies an external cause (whether recorded or not), all injuries (other than sequelae) had to be taken into account in the estimation.

The PMSI data-base uses the ICD-10 diagnostic codes, whereas severity, as defined by the European Commission, is based on the Abbreviated Injury Scale (AIS), requiring a conversion table between the two. This is provided by the AAAM, associating each ICD injury code to an AIS severity grade from 1 (minor) to 6 (maximal). However, this conversion is imprecise, inasmuch as a given ICD-10 code can correspond to several AIS grades. We therefore used a simplified conversion table, associating each ICD-10 injury code to AIS grades in 3 categories: 1 for AIS ≥3, 0 for AIS <3, and 9 when AIS grade is indeterminate.

Other variables were then derived from those of the PMSI: e.g., center location (*Département*), or size and spread of the health center’s activity.

The approach here is per individual and per year rather than per stay. The hash code provided a single anonymous ID for each individual, allowing an individual’s multiple hospital stays to be linked up.

The data has been aggregated at individual level for patients with several stays at hospital within the same year, which occurred in 48% of cases on average. Some variables (hospital stay, number of acts, number of medical unit reports, stay-related group pricing, number of diagnoses) were summed while the maximal value of MAIS was calculated. For other variables (gender, age, type of admission, type of discharge, center location, type of center, size of center, spread of center activity), the first-stay values were kept. However, for anonymisation purpose, the ATIH data did not allow an individual’s first stay to be definitely identified, as several may have the same month of admission. Thus, if several “first” admissions were all in the same month, which was the case for about 4% of patients in the 2010-2017 PMSI-MCO data, the “first” stay was randomly chosen.

To compare our results with those from other European countries, we adhered as closely as possible to the guidelines in Deliverable 7.1 of the SafetyCube project [9, page 59]. Ahead of statistical analysis of the PMSI data, we thus:

- excluded readmissions, by counting individuals rather than stays,
- included short admissions for which stay counted as 0,
- included all ICD-10 diagnoses from S00 to T88 (injury other than sequelae),
- applied correction coefficients for road-users for whom the external cause coded according to ICD-10 was a non-traffic accident.

Our approach here, however, diverged slightly from the recommendation to include all diagnoses with external cause coded on ICD-10 as between V01 and V89 (i.e., all land transport accidents); to these, we added code V99, “Unspecified transport accident”.

In order to estimate the number of seriously injured road users admitted to hospital, we excluded deaths within 30 days of the accident and included patients with serious (MAIS3+) injury who died after 30 days, considered as injured according to road safety injury definition.

The number of road users admitted to hospital dead within 30 days of the accident has also been estimated. The recording of fatalities in police data is considered comprehensive, but does not indicate whether the victims died at the scene of the accident or whether they were transferred to a hospital. For this reason, the exact number of deaths within 30 days among traffic-accident victims admitted to hospital is not known but can be estimated.

**Fig. 1:**
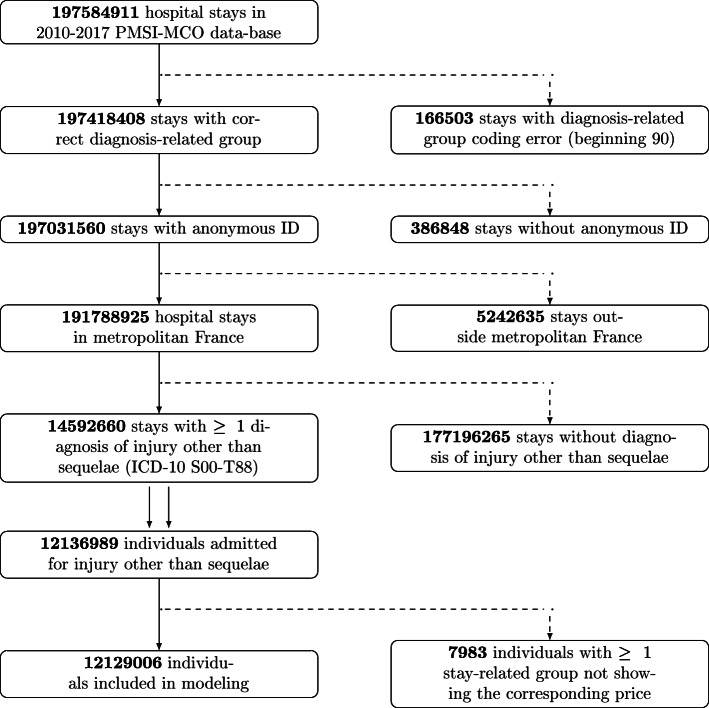
Raw data processing. Diagram showing the processing of the raw 2010-2017 PMSI-MCO data. Dotted arrows show excluded data. Double arrow shows shift from stay-based to individual-based approach

Figure 1 shows the successive stages whereby raw ATIH data were included in the statistical models. **26397538** of the **223982449** anonymous discharge summaries were invoices without hospital stay.

### Estimation method

Victims admitted to hospital after a road accident were identified among all injured patients by the external cause. Unfortunately, as mentioned before, this item was often not recorded. If this external cause was informed randomly, we could consider the subgroup for whom an external cause was specified as being representative of all road-accident casualties admitted to hospital. However, the distributions of rates of recording an external cause versus certain PMSI data showed this not to be the case: reporting rates varied between public and private hospitals and according to many variables in the available data. The recording of an external cause depended on other variables concerning the individual or the health-care establishment.

In practice, for an individual *i* with at least 1 recorded external cause, an estimated correction coefficient $\hat {d}_{i}$ was applied, weighting the individual. The associated estimator $\hat {B}_{R}$ can be written as:

$$\hat{B}_{R}=\sum_{i \in C} \hat{d}_{i}\ r_{i} $$

where *C* is the set of individuals with at least 1 recorded external cause, and$r_{i}=\left \{\begin {array}{ll} 1 & \text {if at least 1 recorded cause for individual}\ i\\& \text { was a road accident};\\ 0 & \text {if not}. \end {array}\right.$

Estimated correction coefficients $\hat {d}_{i}, \forall i \in C$ were calculated by logistic modeling of the relation between recording a cause (or not) and the other variables shown as an additional material [see Additional file 3]. For each individual *i*, this gives an estimate $\hat {p}_{i}$ of the probability of an external cause being reported. The correction coefficients were then obtained by inverting these probabilities [12]: i.e., $\forall i \in C, {\hat {d}_{i}=1/\hat {p}_{i}}$. The associated estimator $\hat {B}_{R}$ can then be written as:

$$\hat{B}_{R}=\sum_{i \in C} \frac{r_{i}}{\hat{p}_{i}} $$

Focusing exclusively on serious (MAIS3+) injury, the estimator can be written as:

$$\hat{B}_{R,3+}=\sum_{i \in C} \frac{m_{i}\ r_{i}}{\hat{p}_{i}} $$

where $m_{i}=\left \{\begin {array}{ll} 1 & \text {if}\ MAIS \geq 3 \text { for individual}\ i;\\ 0 & \text {if}\ MAIS < 3 \text { for individual}\ i. \end {array}\right.$

However, it must be borne in mind that conversion from ICD-10 to AIS is not exact. Many ICD diagnoses have undetermined severity (AIS9). For these missing data, we used simple regression imputation. Another logistic model was fitted from all individuals with determined (yes/no) MAIS3+ status, using the same explanatory variables as before (except for diagnosis-related indicators), here applied to all other individuals. This estimated the probability *P*(*MAIS*_*i*_≥3), denoted as $\hat {m}_{i}$, for all individuals with undetermined severity (MAIS9).

The estimator $\hat {B}_{R,3+}$ is now written as:

$$\hat{B}_{R,3+}=\sum_{i \in C} \frac{\hat{m}_{i}\ r_{i}}{\hat{p}_{i}} $$

with $\hat {m}_{i}=m_{i}$ for individuals with determined MAIS3+ status.

In practice, recording probabilities were calculated on *k*-fold cross-estimation. Derived from cross-validation, this consists in uniform division of the data into *k* samples of comparable size and successive independent estimation of recording probability in each of the *k* samples by constructing the statistical model with the other *k*−1 samples, providing an estimate for each individual. In the present study, we used *k*=100 samples as a compromise between precision and calculation time.

The 4^*th*^ digit of the ICD-10 classification distinguishes traffic and non-traffic accidents. An additional material Table [see Additional file 4] shows ICD-10 codes V01-V89 (Land transport accidents) and V99 (Unspecified transport accident) according to this 4^*th*^ digit.

Studies under the SafetyCube project revealed problems of classifying accidents as traffic/non-traffic, due to differences in how the information is structured in ICD-10 and ICD-9 [9; 13, pages 142-144]. Correction coefficients were proposed. Thus, 97.1% of accidents not involving a motor vehicle (ICD-10 codes V01, V06, V10, V11, V16, V17, V18 and some V80 codes (.0,.1,.2,.7 and.8)) and 61.8% of those involving at least 1 motor vehicle, among accidents classified as non-traffic in ICD-10 were actually traffic accidents. The corrected estimator for admissions following a traffic accident $\hat {B}_{R}^{cor}$ is then written as:

$$\hat{B}_{R}^{cor}=\sum_{i \in RT} \frac{1}{\hat{p}_{i}} + \sum_{i \in NT} \frac{0,971\ (1-h_{i}) + 0,618\ h_{i}}{\hat{p}_{i}} $$

where *RT* represents individuals with recorded external cause as involving a traffic accident, *NT* represents individuals with recorded external cause as involving a non-traffic accident (other than accidents while getting in or out of the vehicle) and $h_{i}=\left \{\begin {array}{ll} 1 & \text {if the external (non-traffic) cause indicates that}\\& \text {individual}\ i\\ & \text {was injured in an accident involving at least 1 motor}\\& \text {vehicle};\\ 0 & \text {if not}. \end {array}\right.$

Focusing exclusively on serious injury (MAIS3+), the estimator is written as:

$$\hat{B}_{R,3+}^{cor}=\sum_{i \in RT} \frac{\hat{m}_{i}}{\hat{p}_{i}} + \sum_{i \in NT} \frac{\hat{m}_{i}\ [0,971\ (1-h_{i}) + 0,618\ h_{i}]}{\hat{p}_{i}} $$

Likewise, we estimated the number of deaths within 30 days among traffic-accident victims admitted to hospital.

Continuous variables were cut into classes and introduced into the logistic model by means of dummy variables. Subgroup analysis required re-estimating the probability of an external cause being recorded, so as to have coherent results. The selection of variables was examined to enhance model quality. The LASSO selection [14] was used at the national and Rhône *Département* levels, based on AIC and BIC criteria. Almost all the variables detailed in the additional material Table [see Additional file 3] were selected. The fact that the rate of registration of the external cause is clearly increasing between 2010 and 2017 led us to build one model per year, which was possible due to the large amount of data.

In order to assess the significance of age effect on severity, we built a log-linear model for male and female separately. We then compared the difference between null and residual deviances to a chi-squared test with 9 degrees of freedom at the 0.05 significance level.

## Results

Estimates of traffic-accident victims admitted to hospital in France between 2010 and 2017 are shown in Table 3 (all severity, and MAIS3+). These estimates exclude cases who died within 30 days of the accident. The number of casualties of whatever severity was stable over the period, at about 100000 per year. Serious injuries ranged from about 18000 in 2010 to 17000 in 2012 and 2013, increasing up to 19768 in 2017, and, as a percentage of all traffic injuries, from 16.9% in 2012 and 2013 to 18.9% in 2017, for a mean 17.5%. The incidence is rather stable, ranging from 158.1 (in 2013) to 167.9 (in 2011) injured road traffic users admitted to hospital per 100000 metropolitan France inhabitants, for a mean of 161.6.

**Table 3:** Number of injured and of seriously injured admitted to hospital

Year	All injuries	MAIS3+ injury	MAIS3+/all injuries	Incidence
				(per 100000 inhabitants)
2010	104917	18116	17.3*%*	167.1
2011	105883	18220	17.2*%*	167.9
2012	100379	16971	16.9*%*	158.4
2013	100737	17024	16.9*%*	158.1
2014	104197	17960	17.2*%*	162.7
2015	101811	17764	17.4*%*	158.3
2016	102192	18518	18.1*%*	158.5
2017	104502	19768	18.9*%*	161.7
Total	824618	144341	17.5*%*	161.6
Average	103077	18043	17.5*%*	161.6

**Fig. 2:**
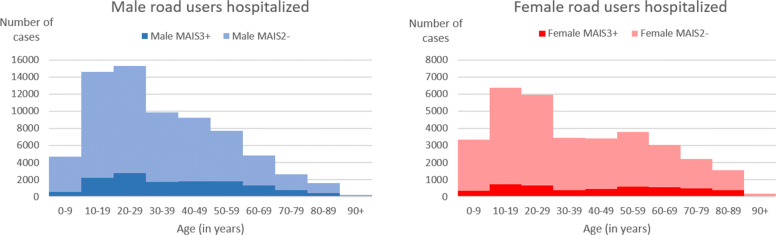
Casualties according to gender, age-group and severity. Average number of cases per year (left, male; right, female) from 2010 to 2017

Figure 2 shows mean estimates of admission following traffic accidents over the period 2010-2017 according to gender (left, male; right, female), age-group and severity (serious, MAIS3+; mild, MAIS2-). As expected, it exhibits a major impact of age and gender, with males on average twice as often admitted to hospital for road accidents (sex ratio, 2.1, *p*-value <10^−4^), and especially among 20-49 year-olds (sex ratio, 2.5, p-value <10^−4^). The sex difference is even sharper in serious injury, at a mean 2.9 (*p*-value <10^−4^) and >4 (*p*-value <10^−4^) for 20-39 year-olds. The age effect in serious injury is highly significant for males victims ($\chi ^{2}_{9}=7266, {p}\text {-value}<10^{-4}$), with serious injury rates ranging from 12% in younger and 31% in older subjects, and for females victims ($\chi ^{2}_{9}=3818, \text {p-value}<10^{-4}$), with serious injury rates ranging from 10% in younger and 24% in older subjects.

One aspect of method validation was to focus on the Rhône area, where the Registry data are available and the real number of MAIS3+ casualties is known. The Rhône road trauma Registry records all road-accident victims presenting to hospital departments, whether admitted or not, with lesion assessment on AIS straightly defined by a trauma coding expert. For purposes of comparison, we converted Registry severity levels for 2010-2014 from AIS98 to AIS2005; data for the subsequent years were already coded in AIS2005. On the other hand for PMSI (with ICD coding featuring no severity scale), we need to approximate the severity using a passage matrix from ICD to AIS. In order to validate our method, we compare the obtained results restricted to the Rhône area with the Rhône road trauma Registry. However, the PMSI data for MAIS3+ admissions in the Rhône *Département* did not specify whether the actual accident had occurred in the area. This mostly explains the differences observed between PMSI estimations and Rhône road trauma Registry records.

As accident location was not part of the PMSI data, we selected in the two data sources only persons residing in the Rhône *Département*, on the assumption that an overwhelming majority of persons both admitted and residing in the Rhône would have had accidents in the same area. In the Registry, 85% of victims were resident in the Rhône *Département*. In that case, we can see that differences between estimations tend to decrease.

Table 4 shows that the PMSI-based estimates largely exceeded the Registry count, by 50% in 2010 and 90% in 2015, but much less so for Rhône residents (right-hand columns), with good agreement for the first 4 years but with overestimation thereafter. This finding incidentally confirms that the Rhône hospitals have a large catchment area: serious and severe (MAIS4+) patients, who are injured nearby, are often transferred to a big and well-equipped hospital in Lyon (located in the Rhône *Département*), which is the biggest city (1,3 millions of inhabitants) in the broader Rhône-Alpes region (8 millions of inhabitants).

**Table 4:** Serious (MAIS3+) casualties on PMSI and Rhône road trauma Registry data, with or without restriction to local residents

	MAIS3+ victims admitted		MAIS3+ victims admitted	
	in the Rhône		and resident in the Rhône	
Year	PMSI	Registry	PMSI	Registry
2010	581	393	319	328
2011	542	386	323	328
2012	557	378	367	306
2013	612	385	362	329
2014	747	407	480	330
2015	724	382	474	318
2016	654	369	427	303
2017	732	419	431	334

Table 5 shows estimated numbers of admitted patients who died within 30 days of a traffic accident between 2010 and 2017. These estimates were compared to police data [15], which is the count of all deaths up to 30 days following a road traffic accident; this collection is exhaustive and includes deaths after hospital admission as well as deaths at the scene. This Table shows that almost two-thirds of subjects who died following a road accident had not been admitted to hospital; i.e., died at the accident scene. However, the proportion of admissions among total deaths tended to decrease, from 36% in 2010 to 27% in 2017.

**Table 5:** Deaths within 30 days of a traffic accident. Annual total, and deaths after hospital admission

Year	Admitted to hospital	All deaths	Ratio deaths after
	(estimates)	(police data)	admission/all deaths
2010	1442	3992	36.1*%*
2011	1356	3963	34.2*%*
2012	1237	3653	33.9*%*
2013	1070	3268	32.7*%*
2014	1049	3384	31.0*%*
2015	1008	3461	29.1*%*
2016	1051	3477	30.2*%*
2017	922	3448	26.7*%*

The number of deaths occurring 30 days after the accident can also be estimated from our hospital data. Thus, the ratio of fatalities after 30 days to total fatalities at 30 days varies between 1.0% and 2.2% depending on the year. In other words, the 30-day limit for the definition of road traffic fatalities excludes only a small proportion of fatalities.

## Discussion

Between 2010 and 2017, about 100000 people were admitted to hospital in France each year following a road accident: i.e., mean incidence, 161.6/100000. As expected, this estimate of 100000 is well above police figures, which range between 26000 and 30000 for the same period. Numbers of serious (MAIS3+) injuries tended to fall between 2010 and 2013, as did the number of deaths, but then strongly increased while mortality remained fairly stable.

Regardless of age, males are more seriously affected than females. This pattern can be seen from childhood, and becomes more marked as subjects begin to use motor vehicles and especially motorized two-wheelers, which are mostly used by males [16]. That serious injury rates increase with age, notably after 70 years, while most victims are either pedestrians or car drivers, is largely due to increasing inherent frailty [17].

As stated in the Background section, in France estimates were hitherto based on other data, with a specific methodology [7]. This is possible because, for a single defined geographical area, we have two accident data sources, the Rhône road trauma Registry and police data, with victims identified in both via probabilistic linking. A capture-recapture method estimates numbers of all victims and MAIS3+ victims in the geographical area, enabling calculation of correction coefficients for under-recording by the police and the resulting bias. Applying these coefficients to police data for France as a whole enables estimation of numbers of all victims and MAIS3+ victims at national level. Comparison between the present and previously reported results, however, is not directly possible, as previous reports did not concern the same years, included serious injury without hospital admission (estimated at 5-10%), and used the AIS90 classification, on which severity is graded at least 10% lower [9, page 159]. However, new estimates will be published shortly by our team that are very similar to those shown in Table 3 once the above differences are taken into account.

The present results for the MAIS3+/mortality ratio can be compared to those in other countries, and especially in Europe, where data also come from hospital discharge registries. For 2014, the ratio in the present data-set is 5.3, compared to 3-4 in Belgium, Italy, Portugal, Spain and Sweden [9, Table 4.5]. Our ratio is slightly high, but in the other countries account was not taken of non-reported external cause, with a rate of about 17% in 2 other countries and not provided in the other 3. There are 2 countries with even lower ratios: 1.7 in Ireland and 0.6 in Poland; the Netherlands and Switzerland reported higher ratios, of 13.2 and 11.9 respectively, but using a different method (capture-recapture with 2 sources: medical and police data) and with large proportions of cyclists, for whom the MAIS3+/mortality ratio is especially high (around 24 in the Netherlands).

The proportion of serious (MAIS3+) injury in victims admitted to hospital is 17% in France as in the UK, but 21% in Germany, 22% in Belgium and 26% in the Netherlands; in Spain, it is 34%, whereas it is only 15% in Sweden and 9% in Austria [8]. These differences in hospital admission policy are doubtless partly due to differences in admission policy.

Relating MAIS3+ injury to population, the ratio is 27.0/100000 in France, similarly to Italy (24.6) and Belgium (26.5). The ratios are lower in Germany (18), Spain (14), Sweden (12) and the UK (8). The Netherlands and Switzerland report higher ratios (44 and 35, respectively), again doubtless due to high rates of cyclists and to the capture-recapture approach.

Concerning mortality, the most interesting statistic is the proportion of deaths before admission (generally, at the accident site). In France, it is the Emergency operator who decides whether to send a medicalized ambulance (SAMU), having assessed the likely severity. According to the Rhône road trauma Registry, more than 90% of immediate deaths were managed by the SAMU, suggesting that reducing immediate mortality is not a matter of improving health response but only of reducing crash severity. The downward trend in 30-day mortality in victims admitted to hospital and in the ratio between deaths following admission and all deaths (Table 5) may be due to improved hospital management of serious injury. Hospital stay costs in case of in-hospital mortality have increased by 158% over the period [18]. Combined with the fall in in-hospital mortality (Table 5), this suggests improved care, in line with the increase in costs per admission over the period.

### Limitations

Estimation quality obviously depends on correction for the high rate of non-recording of an external cause and on severity assessment in these cases.

For correction, we used all the data available in the PMSI records, such as type and size of hospital. Recording of external cause, however, may be linked to other variables not found in the PMSI. On the other hand, the model does have the advantage of being based on a very large number of observations, with 21 indicators and separate yearly assessments so as to be able to adapt to any changes in practices as regards recording the external cause.

For conversion, we used the AAAM10, validated by the AAAM, which is one of the best instruments for converting from ICD-10 to AIS2005 update 2008. Results, however, are approximate, as the two classifications were constructed for different purposes. Several ICD-10 codes may lead to one AIS code or one ICD-10 code to several AIS codes, introducing bias in the estimation of severity.

To validate the method pragmatically, we restricted estimation to the Rhône administrative area for which the observed numbers of serious casualties admitted to hospital were available, the Registry data being almost exhaustive, with lesions directly coded according to AIS by a physician specialized in injury coding. Comparison against this data source was, however, not fully reliable as the PMSI data did not include the administrative area of the accident location (Rhône or neighboring), and the Rhône has a large catchment area: serious and severe patients injured nearby are often transferred to Lyon (located in the Rhône), as it has better hospital infrastructure, being the biggest city in the broader Rhône-Alpes region (8 millions of inhabitants). The fact of having selected the residents of the *Département* mitigates the differences, but does not totally eliminate the disadvantage of not knowing in the PMSI data the *Département* where the accident took place. This clearly appears in Table 4: restriction to Rhône residents considerably reduced differences, although these remained quite high for recent years, and so far we have no explanation for this. Compared to the Rhône road trauma Registry, PMSI estimates are of the same order of magnitude, even if not knowing the accident site precludes real validation. At national level, of course, this problem does not apply, or only to a negligible extent at the country’s borders.

## Conclusion

Using PMSI medico-administrative hospital data allowed estimation of the number of road accident victims admitted to hospital in France between 2010 and 2017 and, among these, the number of seriously injured according to the European Commission definition. This facilitates comparison with other countries using the same kind of hospital data.

To our knowledge, most of hospital discharge data suffer from the same issue, i.e. external cause information often missing in case of trauma. The statistical method applied in our study could then be very efficient to estimate the burden of hospital care for road traffic injuries from medico-administrative data in other countries. In addition, we are currently applying this methodology on the same data to produce estimates of injuries due to other accidents such as home and leisure injuries or occupational injuries.

## Supplementary Information

**Additional file 1** Groups of external causes of morbidity/mortality in ICD-10. This Table shows the different groups of external causes of morbidity/mortality in ICD-10.

**Additional file 2** Rate of recording external causes of morbidity/mortality. This Table contains the computed rate of recording external causes of morbidity/mortality for each year from 2010 to 2017, in percentage of stays for injury other than sequelae.

**Additional file 3** List of logistic model variables. This Table exhaustively lists the explanatory variables included in logistic modeling, with every possible modality for each variable. The last 4 variables characterize the hospital, the others the individual.

**Additional file 4** Distribution of ICD-10 codes of external causes of morbidity/mortality according to 4^*th*^ digit. This Table shows the distribution of ICD-10 codes V01-V89 (Land transport accidents) and V99 (Unspecified transport accident) according to 4^*th*^ digit into 3 modalities: Traffic accident, Non-traffic accident and Accident getting in or out of vehicle.

## Data Availability

The datasets supporting the conclusions of this article are not publicly available. Data access requires a convention between the requesting party and the ATIH, subject to a scientifically justified request and guaranteeing the compliance with data acces instructions. This access is provided from the ATIH website and securised by password and access token. This data access request has also to be validated by the French data protection authority (CNIL).

## Abbreviations

AAAM: American association for automotive medicine
AIS: Abbreviated injury scale
ATIH: Agence technique de l’Information sur l’Hospitalisation [Hospital Information Technical Agency]
ICD: International statistical classification of diseases and related health problems
LASSO: Least absolute shrinkage and selection operator
MAIS: Maximum abbreviated injury scale
MAIS3+: Maximum abbreviated injury scale level 3 or greater
MAIS9: Maximum abbreviated injury scale non-specified
PMSI: Programme de Médicalisation des Systèmes d’Information [French national medico-administrative data-base]
