# Supplementary material for: Estimated number of seriously injured road users admitted to hospital in France between 2010 and 2017, based on medico-administrative data
Source: BMC Public Health. 2021 Mar 8;21:469. doi: 10.1186/s12889-021-10437-0 (PMC7938523; doi:10.1186/s12889-021-10437-0)
Supplement: Supplementary file 1 — Additional file 1 Groups of external causes of morbidity/mortality in ICD-10. This Table shows the different groups of external causes of morbidity/mortality in ICD-10. [file 12889_2021_10437_MOESM1_ESM.pdf]

ICD-10 code	Category
V01-V99	Transport accidents
W00-X59	Other external causes of accidental injury
X60-X84	Intentional self-harm
X85-Y09	Assault
Y10-Y34	Event of undetermined intent
Y35-Y36	Legal intervention and operations of war
Y40-Y84	Complications of medical and surgical care
Y85-Y89	Sequelae of external causes of morbidity and mortality
Y90-Y98	Supplementary factors related to causes of morbidity and mortality classified elsewhere
