# Supplementary material for: Estimated number of seriously injured road users admitted to hospital in France between 2010 and 2017, based on medico-administrative data
Source: BMC Public Health. 2021 Mar 8;21:469. doi: 10.1186/s12889-021-10437-0 (PMC7938523; doi:10.1186/s12889-021-10437-0)
Supplement: Supplementary file 2 — Additional file 2 Rate of recording external causes of morbidity/mortality. This Table contains the computed rate of recording external causes of morbidity/mortality for each year from 2010 to 2017, in percentage of stays for injury other than sequelae. [file 12889_2021_10437_MOESM2_ESM.pdf]

Year	Recording rate
2010	22.35%
2011	24.08%
2012	25.61%
2013	27.23%
2014	29.08%
2015	30.78%
2016	32.81%
2017	33.91%
