# Supplementary material for: Estimated number of seriously injured road users admitted to hospital in France between 2010 and 2017, based on medico-administrative data
Source: BMC Public Health. 2021 Mar 8;21:469. doi: 10.1186/s12889-021-10437-0 (PMC7938523; doi:10.1186/s12889-021-10437-0)
Supplement: Supplementary file 3 — Additional file 3 List of logistic model variables. This Table exhaustively lists the explanatory variables included in logistic modeling, with every possible modality for each variable. The last 4 variables characterize the hospital, the others the individual. [file 12889_2021_10437_MOESM3_ESM.pdf]

Variable	Description	Modalities
sexe	Gender	1(=male) 2(=female)
age_class	Age-group in years	0-9 10-19 20-29 30-39 40-49 50-59 60-69 70-79 80-89 90+
modeEntree	Type of admission	0(=provisional transfer) 6(=intra hospital transfer) 7(=inter hospital transfer) 8(=home)
modeSortie	Type of discharge	0(=provisional transfer) 6(=intra hospital transfer) 7(=inter hospital transfer) 8(=home) 9(=death)
mois	Discharge month	01 02 03 04 05 06 07 08 09 10 11 12
duree_class	Total of all hospital stays (grouped)	0 1 2-7 8-30 31+
Acte_class	Number of classifying acts (grouped)	0 1 2 3-4 5-7 8-15 16+
Rum_class	Number of Medical Unit Reports (grouped)	1 2 3 4+
tarif_GHS_class	Total stay-related group price (grouped)	0-600 600-1000 1000-2000 2000-5000 5000-10000 10000+
Diag_class	Number of diagnoses (grouped)	1 2 3 4-7 8-13 14+
DiagST_class	Number of diagnoses of injury other than sequelae (S00-T88) (grouped)	1 2 3 4+
SejoursST_class	Number of admissions for injury other than sequelae (S00-T88) (grouped)	1 2 3+
Sejours__class	Number of admissions other than for injury other than sequelae (S00-T88) (grouped)	0 1 2 3 4+
ClassAct_C	Indicator of surgical group with classifying procedure	0 1
ClassAct_K	Indicator of surgical group with classifying non-surgical procedure	0 1
ClassAct_M	Indicator of medical group without classifying procedure	0 1
ClassAct_Z	Indicator of undifferentiated group with or without classifying procedure	0 1
S00_S09	Indicator of injuries to the head	0 1
S10_S19	Indicator of injuries to the neck	0 1
S20_S29	Indicator of injuries to the thorax	0 1
S30_S39	Indicator of injuries to the abdomen, lower back, lumbar spine and pelvis	0 1
S40_S49	Indicator of injuries to the shoulder and upper arm	0 1
S50_S59	Indicator of injuries to the elbow and forearm	0 1
S60_S69	Indicator of injuries to the wrist and hand	0 1
S70_S79	Indicator of injuries to the hip and thigh	0 1
S80_S89	Indicator of injuries to the knee and lower leg	0 1
S90_S99	Indicator of injuries to the ankle and foot	0 1
T00_T14	Indicator of injuries involving unspecified or multiple body regions	0 1
T15_T19	Indicator of effects of foreign body entering through natural orifice	0 1
T20_T35	Indicator of burns, corrosions and frosbites	0 1
T36_T65	Indicator of poisoning	0 1
T66_T78	Indicator of other and unspecified effects of external causes	0 1
T79	Indicator of certain early complications of trauma	0 1
T80_T88	Indicator of complications of surgical and medical care, not elsewhere classified	0 1
Dept	Administrative area of hospital	01 02 03 04 05 . . . . 91 92 93 94 95
categ_detail	Detailed category of hospital	Autres_Publics [Other public hospital] CH [Hospital center] CH_ex-CHS [Formerly specialized hospital center] CH_ex-HL [Formerly free hospital center] CHU [University hospital center] CLCC [Cancer center] PSPH/EBNL [Public non-profit hospital] Privé [Private hospital] Privé_non_lucratif [Private non-profit hospital] SIH [Hospital information system]
Taille_class	Size of hospital in annual total number of stays (grouped)	1-10000 10001-15000 15001-30000 30001-100000 100001-200000 200001+
Disp_class	Minimum number of diagnosis-related groups to cover 80% of hospital activity (grouped)	1-60 61-100 101-200 201-300 301+
