# Supplementary material for: Estimated number of seriously injured road users admitted to hospital in France between 2010 and 2017, based on medico-administrative data
Source: BMC Public Health. 2021 Mar 8;21:469. doi: 10.1186/s12889-021-10437-0 (PMC7938523; doi:10.1186/s12889-021-10437-0)
Supplement: Supplementary file 4 — Additional file 4 Distribution of ICD-10 codes of external causes of morbidity/mortality according to 4th digit. This Table shows the distribution of ICD-10 codes V01-V89 (Land transport accidents) and V99 (Unspecified transport accident) according to 4th digit into 3 modalities: Traffic accident, Non-traffic accident and Accident getting in or out of vehicle. [file 12889_2021_10437_MOESM4_ESM.pdf]

ICD-10 code	Traffic accident	Non-traffic accident	Accident getting in or out of vehicle
V01-V06	.1, .9	.0	-
V09	.2, .3	.0, .1, .9	-
V10-V18	.4, .5, .9	.0, .1, .2	.3
V19	.4, .5, .6, .9	.0, .1, .2, .3, .8	-
V20-V28	.4, .5, .9	.0, .1, .2	.3
V29	.4, .5, .6, .9	.0, .1, .2, .3, .8	-
V30-V38	.5, .6, .7, .9	.0, .1, .2, .3	.4
V39	.4, .5, .6, .9	.0, .1, .2, .3, .8	-
V40-V48	.5, .6, .7, .9	.0, .1, .2, .3	.4
V49	.4, .5, .6, .9	.0, .1, .2, .3, .8	-
V50-V58	.5, .6, .7, .9	.0, .1, .2, .3	.4
V59	.4, .5, .6, .9	.0, .1, .2, .3, .8	-
V60-V68	.5, .6, .7, .9	.0, .1, .2, .3	.4
V69	.4, .5, .6, .9	.0, .1, .2, .3, .8	-
V70-V78	.5, .6, .7, .9	.0, .1, .2, .3	.4
V79	.4, .5, .6, .9	.0, .1, .2, .3, .8	-
V80	-	*	-
V81	.1	.0, .2, .3, .5, .6, .7, .8, .9	.4
V82	.1, .9	.0, .2, .3, .5, .6, .7, .8	.4
V83-V86	.0, .1, .2, .3	.5, .6, .7, .9	.4
V87	**	-	-
V88	-	*	-
V89	.2, .3	.0, .1, .9	-
V99	**	-	-

* Codes V80 (Animal-rider or occupant of animal-drawn vehicle injured in transport accident) and V88 (Non-traffic accident of specified type but victim's mode of transport unknown) are always counted as non-traffic.

** Codes V87 (Traffic accident of specified type but victim's mode of transport unknown) and V99 (Unspecified transport accident) are always counted as traffic.
